# Sudden bilateral deafness in a patient with vertebrobasilar artery occlusion: A case report

**DOI:** 10.1097/MD.0000000000036691

**Published:** 2023-12-22

**Authors:** Ying Zhang, Xin Zhao, Ming Zhou, Pengfei Chang, Tao Liu, Yang Li

**Affiliations:** a Department of Encephalopathy, Traditional Chinese Medicine Hospital of Weifang, Weifang, China; b The Second Clinical Medical College, Shandong University of Traditional Chinese Medicine, Jinan, China.

**Keywords:** cerebral infarction, endovascular treatment, sudden bilateral deafness, vertebrobasilar artery occlusion

## Abstract

**Rationale:**

Sudden bilateral deafness is often associated with serious systematic conditions such as neoplasms, vascular events, autoimmune diseases, infections, and iatrogenic injury, but very rarely to cerebrovascular disease. This is a rare case of sudden bilateral deafness in a patient with the vertebrobasilar artery occlusion.

**Patient concerns:**

A 46-year-old man was admitted to a local hospital for sudden bilateral deafness, the patient suffered inarticulate speech and walking unsteadily 6 days later.

**Diagnoses:**

Difusion-weighted magnetic resonance imagin demonstrated acute cerebral infarction in the pons and bilateral cerebellum; Magnetic resonance angiography showed vertebrobasilar artery occlusion.

**Interventions:**

Aspirin and clopidogrel were given for antiplatelet therapy, revascularization was obtained by endovascular treatment.

**Outcomes:**

The symptoms of dysarthria, ataxia and weakness gradually improved and were discharged 14 days after admission revascularization. After 3 months telephone followed-up the patient was self-cared.

**Lessons:**

Deafness sometimes can be an early warning sign of impending vertebrobasilar ischemic stroke. Early recognition of deafness with acute ischemic stroke should allow special management, and misdiagnosis may result in significant morbidity, or even mortality.

## 1. Introduction

Sudden bilateral deafness is often associated with serious systematic conditions such as neoplasms, vascular events, autoimmune diseases, infections, and iatrogenic injury, but very rarely to cerebrovascular disease. However, deafness sometimes can be an early warning sign of impending vertebrobasilar ischemic stroke because the blood supply to the auditory originates from vertebrobasilar system. Early recognition of deafness with acute ischemic stroke should allow special management, and misdiagnosis may result in significant morbidity, or even mortality.

Sudden deafness has been considered traditionally to be a neglected and underestimated symptom of stroke. Because the anterior-inferior cerebellar artery (AICA) which supply the blood to the auditory system originates from the posterior circulation, sometimes patients with cerebral infarction in the posterior circulation can present with sudden deafness.^[1]^ Case reports and case series data suggest patients with sudden deafness should be considered the possibility of vertebrobasilar occlusion, especially for the patients have risk factors for stroke, even if there was no other neurologic signs.^[2–4]^ Because the early diagnosis and proper management may provide a window to prevent the progression of infarction to larger areas, misdiagnosis may result in significant morbidity and mortality. We report a patient suffered bilateral deafness as initial symptoms 6 days prior to permanent infarction.

## 2. Case presentation

**Figure 1.**
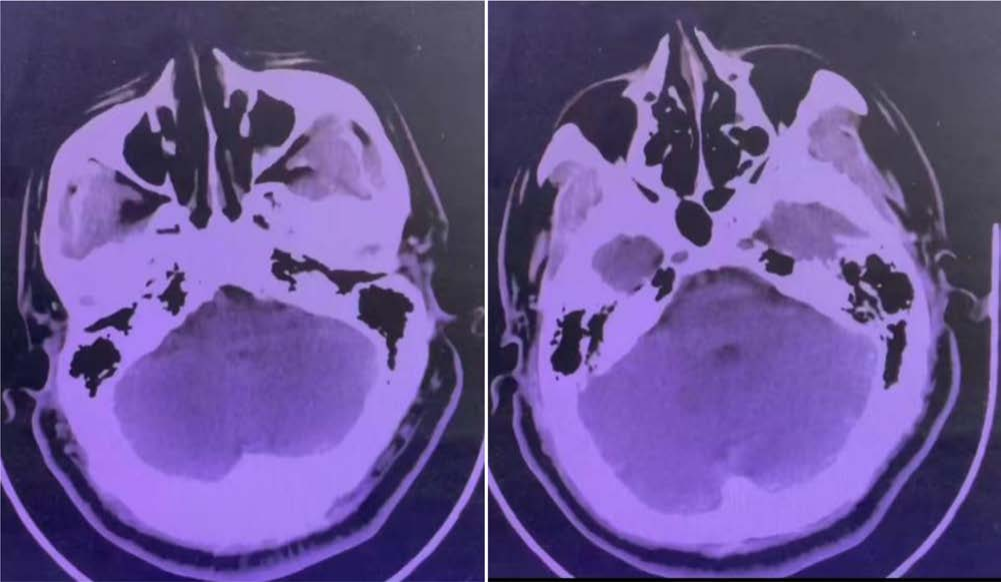
Head computed tomography showed no new lesions in the area of the cerebellar hemispheres and brainstem.

**Figure 2.**
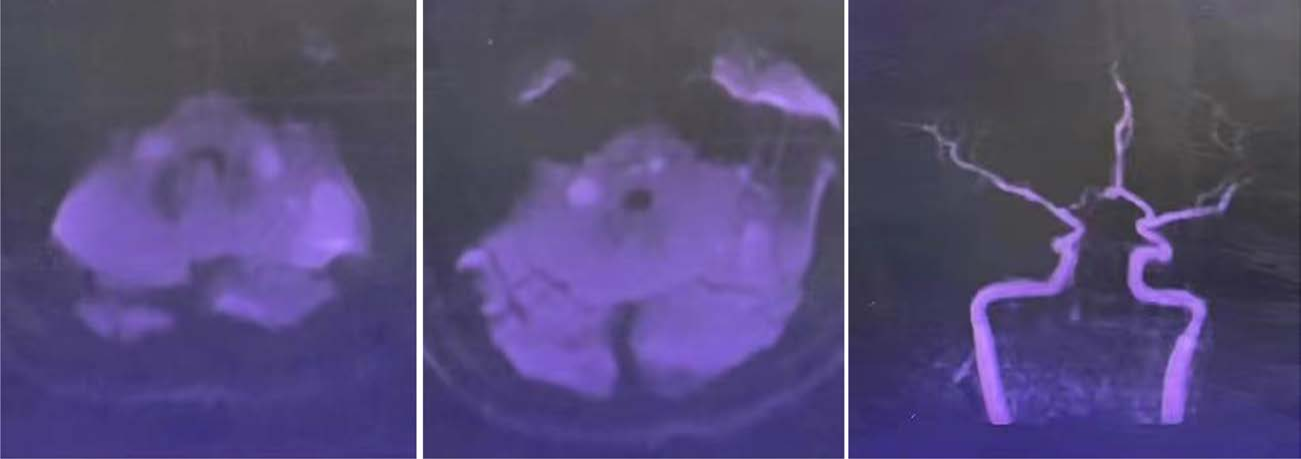
Diffusion-weighted brain MRI showing acute multifocal lesions involving the bilateral cerebellar hemispheres, and the vertebrobasilar artery were occluded on MRA. MRA = magnetic resonance angiography, MRI = magnetic resonance imagin.

A 46-year-old man with a history of hypertension, smoking and alcohol was admitted to local hospital for sudden bilateral deafness, he was diagnosed as sudden deafness and given alprostadil for intravenous injection. His home medications included 20 mg twice daily of nifedipine. His deafness was completely improved. But the patient suffered inarticulate speech and walking unsteadily 6 days later, he was transferred to our department with head computed tomography returned no signifcant fndings (Fig. 1). On admission his neurological examination revealed dysarthria, nystagmus and ataxia, the National Institute of Health Stroke Scale score was 3. His blood pressure was 160/80 mm Hg, his pulse rate was 70 beats/minute, and his temperature was 36.8°C. His general physical examination was unremarkable. Her laboratory test results showed no obvious abnormalities. Difusion-weighted magnetic resonance imagin demonstrated acute cerebral infarction in the pons and bilateral cerebellum; Magnetic resonance angiography showed vertebrobasilar artery occlusion (Fig. 2).

**Figure 3.**
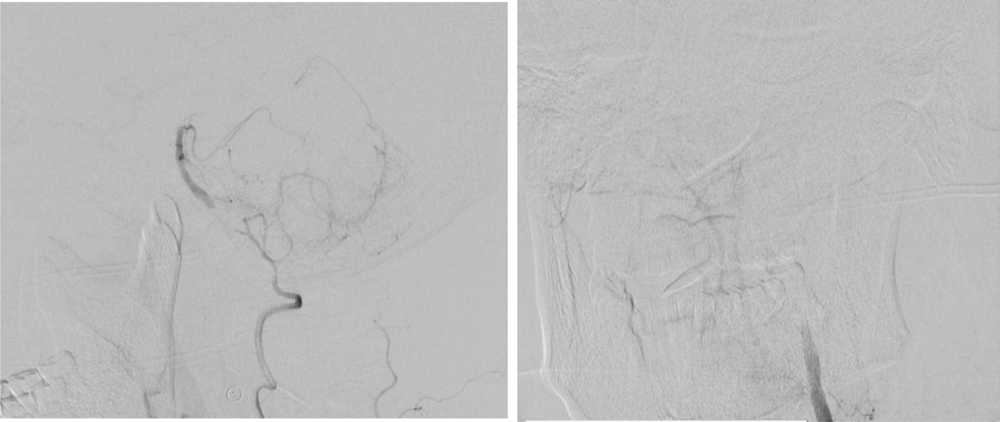
Anteroposterior right vertebral angiography revealed the right vertebral artery was nondominant artery and subtotal occluded in the V4 segment; Anteroposterior left vertebral angiography revealed the left vertebral artery was total occluded in the V2 segment.

**Figure 4.**
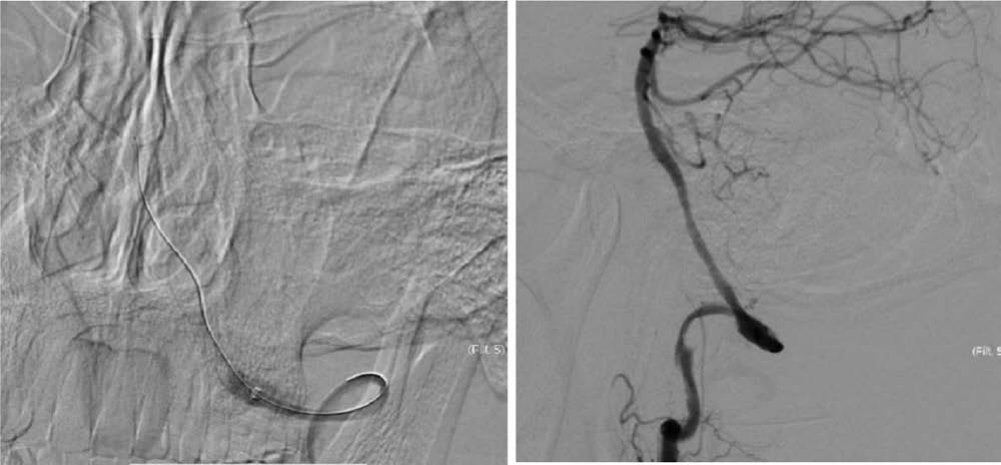
Post-thrombectomy, the last lateralposition left vertebral angiogram demonstrated total recanalization of the BA trunk including the AICA and SCA. AICA = the anterior-inferior cerebellar artery, SCA = superior cerebellar artery.

The delivery of recombinant tissue plasminogen activator (rtPA) has been the standard of care in patients with acute ischemic stroke. However, rtPA must be administered within 4.5h of stroke onset, and it has been 10 hours after the neurologic symptom appeared when he arrived in my hospital. Aspirin and clopidogrel and tirofiban were given for antiplatelet therapy, but his condition was gradually dropped and the strength in his right limb dropped to III level 3 days after his admission. Digital subtraction cerebral angiography showed the right vertebral artery was nondominant artery and subtotal occluded in the V4 segment; the left vertebral artery was total occluded in the V2 segment (Fig. 3). Fortunately, revascularization was obtained by endovascular treatment (Fig. 4), though dysarthria, ataxia and weakness were still remained, he was discharged 14 days after his admission with the the National Institute of Health Stroke Scale scored 7. After 3 months telephone followed-up the patient was self-cared, and the modified rankin scale score was 0.

## 3. Discussion

We report a patient suffered bilateral deafness as initial symptoms 6 days prior to vertebrobasilar system infarction. Fortunately, revascularization was obtained by endovascular treatment and his physical condition was gradually improved. It suggested that deafness sometimes can be an early warning sign of impending vertebrobasilar ischemic stroke. Early recognition of deafness with acute ischemic stroke is very important, because vertebrobasilar ischemia is life threatening and proper management may provide a window to prevent the progression of infarction to larger areas.

Sudden-onset deafness is often due to otolaryngologic and very rarely to cerebrovascular disease. However, because AICA which supply the blood to the auditory system originates from the posterior circulation, sometimes patients with cerebral infarction in the posterior circulation can present with sudden deafness.^[5]^ Usually the internal auditory artery (IAA) which originates from the AICA is an end artery with minimal collaterals and especially vulnerable to ischemia.^[6–8]^ Because the labyrinth requires high-energy metabolism and receives its sole supply from the IAA, the labyrinth is especially vulnerable to ischemia. Sometimes, patients with anterior-inferior cerebellar artery infarction have isolated fluctuating hearing loss, or tinnitus as initial symptoms 1 to 10 days prior to permanent infarction.^[9–11]^

At the same time, AICA constantly supplies the peripheral vestibular structures such as the inner ear and vestibulocochlear nerve, in addition to the central vestibular structures.^[12]^ As a result, in contrast to other cerebellar artery territory infarction, complete AICA infarction usually results in combined peripheral and central vestibular damages.^[13,14]^ Identifying stroke among patients presenting with deafness is one of the most challenging issues in neurology and emergency medicine. Sudden deafness in patients with risk factors of cerebrovascular disease should be prioritized for stroke workup.^[15,16]^

At the time of admission to his local hospital, the patient in our case presented with only bilateral hearing loss without any other neurological deficits, consequently, initially he was not suspected specifc anomalies including vertebrobasilar impairment. Because the inner ear requires a high-energy metabolism and the IAA is an end artery with little collateral circulation, the inner ear is particularly vulnerable to ischemia.^[10–14]^ Deafness sometimes is an early warning sign of impending vertebrobasilar ischemic stroke. The rtPA has been the standard of care in patients with acute ischemic stroke. However, it has been 10 hours after the neurologic symptom appeared. Though antiplatelet therapy was given, his condition was gradually dropped. Vertebrobasilar ischemia is life threatening and poorly outcome, endovascular treatment was recommended for patients with symptomatic non-acute vertebrobasilar artery occlusion.^[17–19]^ Fortunately, though the patient had dysarthria, ataxia and weakness, the condition was gradually improved after the revascularization by endovascular treatment.

Although deafness can present as a sign of AICA infarction, the incidence differs from a low of 30 % to a high of 100 %.^[20]^ There were 2 factors inclued: on the 1 hand neurologists have not included the audiogram as a routine diagnostic tool for the AICA infarction; on the other hand, patients might not be aware of the hearing loss during an attack of vertigo when the unilateral hearing loss is mild or the vertigo is severe.^[21]^

A recent study^[22]^ on the long-term outcome suggest that recovery of hearing loss of a vascular cause is more common than previously thought, approximately 65 % of the patients due to posterior circulation ischemic stroke showed a partial or complete hearing recovery followed for at least 1 year after the onset.^[23]^

## 4. Conclusion

This is a rare case of sudden bilateral deafness in a patient with the vertebrobasilar artery occlusion. The delayed diagnosis of ischemic stroke in the posterior circulation might be poorly outcome and life threatening. Fortunately, our case had a good outcome after the revascularization by endovascular treatment. The early diagnosis and the proper management may provide a window to prevent the progression of infarction to larger areas. We emphasis that the possibility of vertebrobasilar occlusive disorder should be considered for patients with sudden deafness especially those with risk factors for stroke, even though no neurological signs were found.

## Author contributions

**Conceptualization:** Ying Zhang, Xin Zhao, Ming Zhou, Tao Liu, Yang Li.

**Data curation:** Ying Zhang, Xin Zhao, Pengfei Chang, Tao Liu, Yang Li.

**Formal analysis:** Ying Zhang, Xin Zhao, Ming Zhou, Tao Liu, Yang Li.

**Funding acquisition:** Ying Zhang, Pengfei Chang.

**Investigation:** Ying Zhang, Ming Zhou.

**Methodology:** Ying Zhang, Tao Liu, Yang Li.

**Project administration:** Ying Zhang, Ming Zhou, Pengfei Chang, Yang Li.

**Resources:** Ying Zhang, Pengfei Chang, Tao Liu, Yang Li.

**Software:** Ying Zhang, Pengfei Chang, Yang Li.

**Supervision:** Yang Li.

**Validation:** Yang Li.

**Visualization:** Yang Li.

**Writing – original draft:** Ying Zhang, Ming Zhou, Pengfei Chang, Yang Li.

**Writing – review & editing:** Ying Zhang, Tao Liu, Yang Li.

## References

[1] Kim HA Lee H. Recent advances in understanding audiovestibular loss of a vascular cause. J Stroke. 2017;19:61–6. 28030893 10.5853/jos.2016.00857 PMC5307938

[2] Tomoya K1 Keisuke I Shinichi U. Basilar artery occlusion presenting as sudden bilateral deafness: a case report. J Med Case Reports 2021;15:111. 10.1186/s13256-020-02574-8 PMC7927263 33653404

[3] Caroline EP Christina FB Helle KI. Sudden bilateral deafness in a patient with transient ischemic attack: a case report. Case Rep Neurol. 2021;13:119–22. 33790769 10.1159/000512403 PMC7989795

[4] Xinlin W Minsun Xiaoping W. Cerebellar artery infarction with sudden hearing loss and vertigo as initial symptoms: a case report. World J Clin Cases. 2021;9:2519–23. 33889616 10.12998/wjcc.v9.i11.2519 PMC8040184

[5] Ji Soo K Hyung L. Inner ear dysfunction due to vertebrobasilar ischemic stroke. Semin Neurol. 2009;29:534–40. 19834865 10.1055/s-0029-1241037

[6] Perlman HB Kimura RS Fernandez C. Experiments on temporary obstruction of the internal auditory artery. Laryngoscope. 1959;69:591–613. 13673604 10.1288/00005537-195906000-00001

[7] Grad A Baloh RW. Vertigo of vascular origin. Clinical and electronystagmographic features in 84 cases. Arch Neurol. 1989;46:281–4. 2919982 10.1001/archneur.1989.00520390047014

[8] Oas JG Baloh RW. Vertigo and the anterior inferior cerebellar artery syndrome. Neurology. 1992;42:2274–9. 1461378 10.1212/wnl.42.12.2274

[9] Yi HA Lee SR Lee H. Sudden deafness as a sign of stroke with normal diffusionweighted brain MRI. Acta Otolaryngol. 2005;125:1119–21. 16298797 10.1080/00016480510033676

[10] Lee H Kim HJ Koo JW. Progression of acute cochleovestibulopathy into anterior inferior cerebellar artery infarction. J Neurol Sci. 2009;278:119–22. 19108852 10.1016/j.jns.2008.11.019

[11] Kim JS Cho KH Lee H. Isolated labyrinthine infarction as a harbinger of anterior inferior cerebellar artery territory infarction with normal diffusion-weighted brain MRI. J Neurol Sci. 2009;278:82–4. 19135217 10.1016/j.jns.2008.12.002

[12] Lee H Sohn SI Jung DK. Sudden deafness and anterior inferior cerebellar artery infarction. Stroke. 2002;33:2807–12. 12468774 10.1161/01.str.0000038692.17290.24

[13] Amarenco P Hauw JJ. Cerebellar infarction in the territory of the anterior and inferior cerebellar artery. A clinicopathological study of 20 cases. Brain. 1990;113 (Pt 1)(Pt 1):139–55. 2302529 10.1093/brain/113.1.139

[14] Amarenco P Rosengart A DeWitt LD. Anterior inferior cerebellar artery territory infarcts. Mechanisms and clinical features. Arch Neurol. 1993;50:154–61. 8431134 10.1001/archneur.1993.00540020032014

[15] Tzu-Pu C Zheyu W Ariel A. Sudden hearing loss with vertigo portends greater stroke risk than sudden hearing loss or vertigo alone. J Stroke Cerebrovasc Dis. 2017;09:033. 10.1016/j.jstrokecerebrovasdis.2017.09.033 PMC6049697 29102540

[16] Newman-Toker DE. Missed stroke in acute vertigo and dizziness: it is time for action, not debate. Ann Neurol. 2016;79:27–31. 26418192 10.1002/ana.24532 PMC9041814

[17] Berkhemer OA Fransen PS Beumer D. A randomized trial of intraarterial treatment for acute ischemic stroke. N Engl J Med. 2015;372:11–20. 25517348 10.1056/NEJMoa1411587

[18] Saver JL Goyal M Bonafe A. Stent-retriever thrombectomy after intravenous t-PA vs t-PA alone in stroke. N Engl J Med. 2015;372:2285–95. 25882376 10.1056/NEJMoa1415061

[19] Nogueira RG Jadhav AP Haussen DC. Thrombectomy 6 to 24 hours after stroke with a mismatch between defcit and infarct. N Engl J Med. 2018;378:11–21. 29129157 10.1056/NEJMoa1706442

[20] Hyun-Ah K Hyon-Ah Y Hyung L. Recent advances in cerebellar ischemic stroke syndromes causing vertigo and hearing loss. Springer. 2015;17:11. 10.1007/s12311-015-0745-x 26573627

[21] Cnyrim CD Newman-Toker D Karch C. Bedside differentiation of vestibular neuritis from central Bvestibular pseudoneuritis^. J Neurol Neurosurg Psychiatry. 2008;79:458–60. 18344397 10.1136/jnnp.2007.123596

[22] Kim HA Lee BC Hong JH. Longterm prognosis for hearing recovery in stroke patients presenting vertigo and acute hearing loss. J Neurol Sci. 2014;339:176–82. 24581671 10.1016/j.jns.2014.02.010

[23] Lee H Yi HA Chung IS. Long-term outcome of canal paresis of a vascular cause. J Neurol Neurosurg Psychiatry. 2011;82:105–9. 20587486 10.1136/jnnp.2009.180497

